# A Difference-in-Differences Analysis of Youth Smoking and a Ban on Sales of Flavored Tobacco Products in San Francisco, California

**DOI:** 10.1001/jamapediatrics.2021.0922

**Published:** 2021-05-24

**Authors:** Abigail S. Friedman

**Affiliations:** 1 Department of Health Policy and Management, Yale School of Public Health, New Haven, Connecticut

## Abstract

This difference-in-differences analysis compared San Francisco, California, with 7 other districts in California, Florida, New York, and Pennsylvania to explore the association of a policy completely banning flavored tobacco with tobacco use.

Restrictions on flavored tobacco product sales are increasingly popular; 5 US states and hundreds of localities have implemented them in the past few years alone. Yet only 1 study,^1^ to my knowledge, has considered how complete flavor bans applying to electronic nicotine delivery systems and combustible tobacco products, without retailer exemptions, are associated with tobacco use. A convenience sample of residents of San Francisco, California, aged 18 to 34 years who had ever used a tobacco product showed significant reductions in any tobacco use following the city’s flavor ban, with a marginally significant increase in combustible cigarette use (smoking) among those aged 18 to 24 years.^1^ Absent a comparison group, however, it is impossible to ascertain if preexisting trends could have driven these findings.

Given the relative health costs of smoking vs vaping nicotine,^2,3^ flavor bans that increase smoking may prove harmful. Thus, this study’s objective was to estimate the association between San Francisco’s ban on flavored tobacco product sales and smoking among high school students younger than 18 years.

## Methods

Data came from the 2011-2019 Youth Risk Behavior Surveillance System (YRBSS) biennial school district surveys, with consideration restricted to districts with representative smoking data (with response rates ≥60%) available through the US Centers for Disease Control and Prevention for each wave: New York City, New York; Broward County, Florida; Los Angeles, California; Orange County, Florida; Palm Beach County, Florida; Philadelphia, Pennsylvania; and San Diego, California, as well as San Francisco, California. This analysis focused on high school students younger than 18 years who had nonmissing data for the outcome of interest: a binary indicator for recent (ie, past 30-day) smoking. This study was deemed exempt from institutional review board review under US federal regulation 45 CFR 46.101(b)(4). The analysis used publicly available YBRSS data, a survey with collection procedures designed to maintain student anonymity; therefore, informed consent was not required.

A binary exposure variable captured whether a complete ban on flavored tobacco product sales was in effect in the respondent’s district on January 1 of the survey year. (The YRBSS is fielded during the spring semester and does not report interview dates; further details are in the eMethods in the Supplement.)

Recent vaping was not considered because of likely confounding. California legalized recreational marijuana use the same year San Francisco’s flavor ban went into effect; in addition, the YRBSS’s vaping questions did not distinguish vaping nicotine vs marijuana.

Covariates captured age, sex, and race/ethnicity fixed effects and tobacco policies on January 1 of the survey year (specifically, state-plus-district conventional cigarette taxes and indicators for smoke-free restaurant laws). San Francisco did not implement other new tobacco control policies between the 2017 and 2019 surveys.^4^

 To compare trends, annual sample-weighted means and 95% CIs were plotted for recent smoking in San Francisco vs other districts. Difference-in-differences analyses used logistic regressions to estimate changes in recent smoking in San Francisco relative to other districts, before vs after the flavor ban’s implementation, adjusting for year and district fixed effects alongside the aforementioned demographic and policy covariates. Robustness checks further adjusted for district-specific time trends and considered California districts only, to ensure uniform state policy exposure. Two-tailed *P* values less than .05 were considered significant. Data were analyzed from February 2021 to March 2021 using Stata version 14 (StataCorp).

## Results

**Figure 1.**
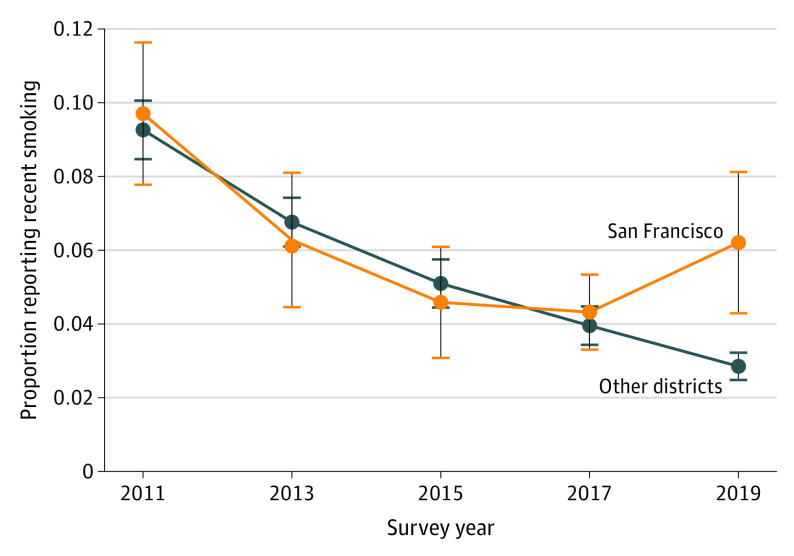
Past-30-Day Smoking Trends Among High School Students Younger Than 18 Years Adjusting for complex survey design, annual, sample-weighted recent smoking rates and their 95% CIs were plotted using district-level Youth Risk Behavior Surveillance System data on recent smoking in high school students younger than 18 years in San Francisco, California, vs 7 other districts with representative data in 2011, 2013, 2015, 2017, and 2019: Broward County, Florida; Los Angeles, California; New York City, New York; Orange County, Florida; Palm Beach County, Florida; Philadelphia, Pennsylvania; and San Diego, California.

**Figure 2.**
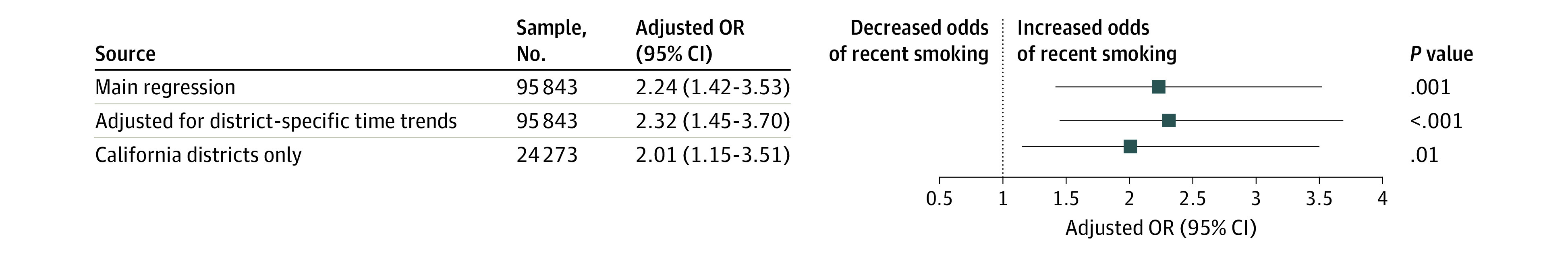
San Francisco’s Ban on Flavored Tobacco Product Sales and Youth Smoking: Difference-in-Differences Estimates Adjusted odds ratios (ORs) and 95% CIs describe difference-in-differences estimates for the association between the ban in San Francisco, California, on flavored tobacco product sales and youth smoking. Specifically, sample-weighted logistic regressions compare youth smoking in San Francisco before vs after its ban on sales of flavored tobacco products went into effect, with concurrent trends in smoking among respondents in the other sites (a difference-in-differences research design). Analyses use 2011-2019 Youth Risk Behavior Surveillance System data on minor respondents from 8 districts: Broward County, Florida; Los Angeles, California; New York City, New York; Orange County, Florida; Palm Beach County, Florida; Philadelphia, Pennsylvania; San Diego, California; and San Francisco, California. The robustness check of California districts only limited consideration to districts in that state. All regressions were adjusted for demographic covariates (age, sex, and race/ethnicity fixed effects), the conventional cigarette tax rate, and a binary indicator for whether the district had a smoke-free restaurant law at a given wave, as well as year and district fixed effects. Analyses were adjusted for complex survey design.

The data set yielded an analytic sample of 100 695 minors, 95 843 of whom had nonmissing data on recent smoking. Among those with data, 9225 respondents came from San Francisco vs 86 618 from other districts, with weighted means indicating smoking rates of 6.2% (95% CI, 5.2%-7.1%) and 5.6% (95% CI, 5.3%-5.9%), respectively. Comparing recent smoking rates by wave revealed similar trends in San Francisco vs other districts prior to 2018 but subsequent divergence (2019: San Francisco, 6.2% [95% CI, 4.2%-8.2%]; other districts, 2.8% [95% CI, 2.4%-3.1%]; Figure 1). Difference-in-differences analyses found that San Francisco’s flavor ban was associated with more than doubled odds of recent smoking among underage high school students relative to concurrent changes in other districts (adjusted odds ratio, 2.24 [95% CI, 1.42-3.53]; *P* = .001; Figure 2). This result was robust to adjustment for district-specific time trends (adjusted odds ratio, 2.32 [95% CI, 1.45-3.70]; *P* < .001) and limiting consideration to California (adjusted odds ratio, 2.01 [95% CI, 1.15-3.51]; *P* = .01).

## Discussion

San Francisco’s ban on flavored tobacco product sales was associated with increased smoking among minor high school students relative to other school districts. While the policy applied to all tobacco products, its outcome was likely greater for youths who vaped than those who smoked due to higher rates of flavored tobacco use among those who vaped.^5^ This raises concerns that reducing access to flavored electronic nicotine delivery systems may motivate youths who would otherwise vape to substitute smoking. Indeed, analyses of how minimum legal sales ages for electronic nicotine delivery systems are associated with youth smoking also suggest such substitution.^6^

This study’s primary limitation is generalizability. Future research should assess whether estimates hold over time and in other localities and consider how policy heterogeneity (eg, retailer exemptions) modifies such bans’ outcomes.

## Notes

Supplement. eMethods.
