# Supplementary material for: A Difference-in-Differences Analysis of Youth Smoking and a Ban on Sales of Flavored Tobacco Products in San Francisco, California
Source: JAMA Pediatr. 2021 May 24;175(8):863–5. doi: 10.1001/jamapediatrics.2021.0922 (PMC8145156; doi:10.1001/jamapediatrics.2021.0922)
Supplement: Supplement. — eMethods. [file jamapediatr-e210922-s001.pdf]

Supplemental Online Content

Friedman AS. A Difference-in-differences analysis of youth smoking and a ban on sales of flavored tobacco products in San Francisco, California. *JAMA Pediatr*. Published online May 24, 2021. doi:10.1001/jamapediatrics.2021.0922

eMethods.

This supplemental material has been provided by the authors to give readers additional information about their work.

© 2021 American Medical Association. All rights reserved.

eMethods.

I. Data and Methods

Data came from the 2011-2019 Youth Risk Behavior Surveillance System's (YRBSS) biennial school district surveys. Among the district-level data available through the Centers for Disease Control and Prevention (CDC), 9 districts had representative data for all 5 waves (i.e., response rates ≥ 60%). One of these districts omitted the past-month smoking question in two waves, and thus was not considered. Thus, data from the following 8 districts were included in this study's analyses: Broward County, FL; Los Angeles, CA; New York City, NY; Orange County, FL; Palm Beach County, FL; Philadelphia, PA; San Diego, CA; and, San Francisco, CA.

The YRBSS's binned age-variable does not distinguish exact year of age for students 18 and older. This precludes identifying whether a particular respondent in the YRBSS's highest age-bin can legally purchase tobacco products in a given year, since many states and localities adopted minimum legal purchasing ages of 19 or 21 between 2011 and 2019. Restricting consideration to minors sidesteps this issue, as prior quasi-experimental work has found substantive effects of tobacco-21 laws on smoking among 18, 19, and 20 year-olds, but statistically insignificant results for minors.[1]

The final analytic sample covered 100,695 respondents, 95,843 of whom had non-missing data for past-30-day smoking. Using binary outcome (recent smoking) and exposure (flavor ban) indicators, the regression specification was: $RecentSmoker_{idt} = \beta_0 + \beta_1 Ban_{idt} + \lambda\overrightarrow{X_{idt}} + \gamma_d + \delta_t + \varepsilon_{itd}$, where subscripts refer to individual *i*, in district *d*, at survey year *t*. The vector of control variables ($\overrightarrow{X_{idt}}$) includes fixed effects for age, sex, and YRBSS race/ethnicity categories[2], as well as state-plus-district conventional cigarette taxes and a binary indicator for smoke-free restaurant laws, as of January 1st of the survey year. (YRBSS surveys are fielded in the spring semester. The data do not report interview dates.) Logistic regressions were sample-weighted and adjusted for complex survey design.

The first robustness check further adjusts for linear time-trends by district, to ensure that the flavor ban's coefficient is not drive by differences in local trends. The second robustness check assesses the main specification regression for California-districts only, to ensure that unobserved state policy variation was not driving results (i.e., since both San Francisco and comparison districts in California would have been exposed to the same state policies).

---

1 Bryan C, Hansen B, McNichols D, and Sabia JJ (2020). Do State Tobacco 21 Laws Work? NBER Working Paper # 28173. Available from: <http://www.nber.org/papers/w28173>.

2 Race/ethnicity categories were based on YRBSS's most detailed race variable ("race7"): White, American Indian/Alaska Native, Asian, Black, Hispanic, Native Hawaiian/Other Pacific Islander, Non-Hispanic with multiple races, and race-missing.

© 2021 American Medical Association. All rights reserved.
